# Effects of Antibiotic Pretreatment of an Ulcerative Colitis-Derived Fecal Microbial Community on the Integration of Therapeutic Bacteria *In Vitro*

**DOI:** 10.1128/mSystems.00404-19

**Published:** 2020-01-28

**Authors:** Kaitlyn Oliphant, Kyla Cochrane, Kathleen Schroeter, Michelle C. Daigneault, Sandi Yen, Elena F. Verdu, Emma Allen-Vercoe

**Affiliations:** a Department of Molecular and Cellular Biology, University of Guelph, Guelph, Ontario, Canada; b Division of Gastroenterology, Department of Medicine, Farncombe Family Digestive Health Institute, McMaster University, Hamilton, Ontario, Canada; University of California, San Diego

**Keywords:** antibiotic pretreatment, bioreactor, fecal microbiota transplantation, human gut microbiome, microbial ecosystem therapeutics, rifaximin, *in vitro* model

## Abstract

Fecal microbiota transplantation (FMT) is a proposedly useful strategy for the treatment of gastrointestinal (GI) disorders through remediation of the patient gut microbiota. However, its therapeutic success has been variable, necessitating research to uncover mechanisms that improve patient response. Antibiotic pretreatment has been proposed as one method to enhance the success rate by increasing niche availability for introduced species. Several limitations hinder exploring this hypothesis in clinical studies, such as deleterious side effects and the development of antimicrobial resistance in patients. Thus, the purpose of this study was to evaluate the use of an *in vitro*, bioreactor-based, colonic ecosystem model as a form of preclinical testing by determining how pretreatment with the antibiotic rifaximin influenced engraftment of bacterial strains sourced from a healthy donor into an ulcerative colitis-derived defined microbial community. Distinct species integrated under the pretreated and untreated conditions, with the relative rifaximin resistance of the microbial strains being an important influencer. However, both conditions resulted in the integration of taxa from *Clostridium* clusters IV and XIVa, a concomitant reduction of *Proteobacteria*, and similar decreases in metabolites associated with poor health status. Our results agree with the findings of similar research in the clinic by others, which observed no difference in primary patient outcomes whether or not patients were given rifaximin prior to FMT. We therefore conclude that our model is useful for screening for antibiotics that could improve efficacy of FMT when used as a pretreatment.

**IMPORTANCE** Patients with gastrointestinal disorders often exhibit derangements in their gut microbiota, which can exacerbate their symptoms. Replenishing these ecosystems with beneficial bacteria through fecal microbiota transplantation is thus a proposedly useful therapeutic; however, clinical success has varied, necessitating research into strategies to improve outcomes. Antibiotic pretreatment has been suggested as one such approach, but concerns over harmful side effects have hindered testing this hypothesis clinically. Here, we evaluate the use of bioreactors supporting defined microbial communities derived from human fecal samples as models of the colonic microbiota in determining the effectiveness of antibiotic pretreatment. We found that relative antimicrobial resistance was a key determinant of successful microbial engraftment with rifaximin (broad-spectrum antibiotic) pretreatment, despite careful timing of the application of the therapeutic agents, resulting in distinct species profiles from those of the control but with similar overall outcomes. Our model had results comparable to the clinical findings and thus can be used to screen for useful antibiotics.

## Abstract (precis)

Patients with gastrointestinal disorders often exhibit derangements in their gut microbiota, which can exacerbate their symptoms. Replenishing these ecosystems with beneficial bacteria through fecal microbiota transplantation is thus a proposedly useful therapeutic; however, clinical success has varied, necessitating research into strategies to improve outcomes. Antibiotic pretreatment has been suggested as one such approach, but concerns over harmful side effects have hindered testing this hypothesis clinically. Here, we evaluate the use of bioreactors supporting defined microbial communities derived from human fecal samples as models of the colonic microbiota in determining the effectiveness of antibiotic pretreatment. We found that relative antimicrobial resistance was a key determinant of successful microbial engraftment with rifaximin (broad-spectrum antibiotic) pretreatment, despite careful timing of the application of the therapeutic agents, resulting in distinct species profiles from those of the control but with similar overall outcomes. Our model had results comparable to the clinical findings and thus can be used to screen for useful antibiotics.

## INTRODUCTION

The consortium of microorganisms that populate the human gastrointestinal (GI) tract, referred to as the gut microbiota, is known to be a critical component of human health, contributing to essential processes, including the metabolism of otherwise indigestible foods to produce nutrients (1, 2) and immune system modulation (3). Multiple GI disorders are associated with derangements in colonic ecosystem composition and function, loosely referred to as dysbiosis (4, 5). Whether dysbiosis is a cause or an effect of disease remains to be elucidated in most cases; however, procedures that aim to ameliorate dysbiosis through the modulation of microbial abundances are nonetheless currently being studied. Among these methods are the use of antibiotics, to deplete opportunistic pathogens, and fecal microbiota transplantation (FMT), to replenish beneficial microbes, which entails the transfer of microbes directly from stool provided by a prescreened donor into a patient (4, 5). The efficacy of these therapies in treating such GI disorders, including irritable bowel syndrome (IBS) (6, 7) and inflammatory bowel diseases (IBD) (8–10), overall has been mixed. Thus, focus has now shifted toward developing an understanding of the factors contributing to the success of these therapies.

Recently, a meta-analysis conducted by Keshteli et al. concluded that antibiotic pretreatment improved the clinical response rate of FMT in ulcerative colitis (UC) patients, a form of IBD, by about 20% (11). The rationale behind this observation was that antibiotics “clear niches and therefore create space” for the donor microbes. However, no study has definitively demonstrated such niche clearing to date. The proposed benefit suggests that this is a hypothesis worth exploring, but there are several barriers to conducting an investigation to specifically look at such an effect in clinical trials. For example, antibiotics can have deleterious side effects; for the most commonly prescribed antibiotics in IBD (ciprofloxacin and metronidazole), these include tendonitis, photosensitivity, birth defects, oral thrush, QT prolongation, GI disturbances, and peripheral neuropathy (long-term administration), in addition to the risks of antibiotic-associated infection (e.g., caused by Clostridioides difficile) and antimicrobial resistance (10). These concerns, coupled with an inconsistent and unsubstantiated benefit, are why antibiotic use is currently not recommended as a treatment option for complex GI disorders such as IBD (12–14). Therefore, few clinical studies have been conducted on the use of antibiotics as a pretreatment for FMT in IBD, and almost none have employed a control arm with no antibiotic pretreatment (11, 15). There are also many questions surrounding how to best apply an antibiotic pretreatment for IBD prior to FMT, such as deciding which antibiotics to use, the dosage, and the timing, in addition to determining for which clinical indications such treatment would prove to be beneficial. Clearly, it would be advantageous to screen for the likely success of various treatment strategies before moving to the clinic.

One study that evaluated the use of antibiotics as a pretreatment for FMT in UC patients and included a relevant control arm was conducted by El-Nachef et al. and focused on the use of rifaximin (16). Rifaximin is a rifamycin class antibiotic that inhibits bacterial DNA-dependent RNA synthesis and is attractive as a therapy because its nonabsorbable nature increases safety compared to use of conventional broad-spectrum antibiotics (16–18). Rifaximin implementation has also shown some success in treating the symptoms of UC (17–20). In their study, El-Nachef et al. found that there was some improvement in secondary outcomes in terms of levels of fecal calprotectin, C-reactive protein (CRP), and abdominal pain with rifaximin pretreatment compared to results with no pretreatment prior to FMT (16). However, the authors did not analyze the gut microbiota of the recipients, and thus the effect of rifaximin on any changes in the gut microbiota before and after FMT were not measured. Therefore, we conducted a pilot *in vitro* study to determine if applying rifaximin as a pretreatment to a UC-derived microbial ecosystem would increase the incorporation of allochthonous microbes sourced from a healthy donor and formulated into a microbial ecosystem therapeutic (MET) for addition to the UC-derived ecosystem. In order to specifically control for environmental factors and allow focus on microbial changes, we utilized bioreactors as models of the distal human gut, populated with well-defined but complex microbial communities. This approach not only allowed strict control over confounding parameters that are prevalent in human studies but also enabled us to gain insight into the mechanistic determinants of integration through strain characterization and deeper analysis. We additionally aimed to determine how results with this *in vitro* approach compared to clinical findings in order to evaluate the usefulness of our method as a form of preclinical testing of the effects of antibiotic pretreatment in microbial replenishment therapies such as FMT and MET.

## RESULTS

### Distinct bacteria incorporated into rifaximin-pretreated versus untreated communities.

**FIG 1:**
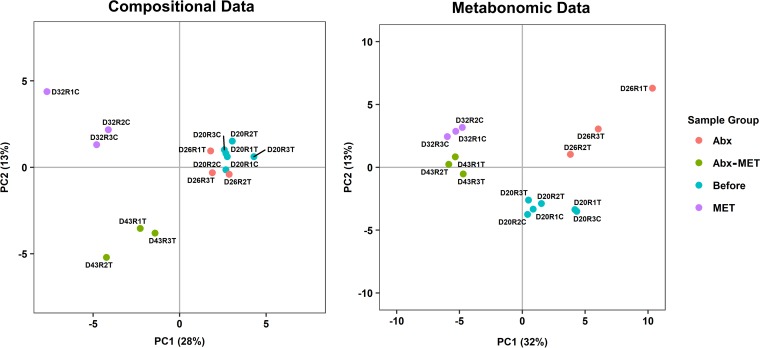
Partial least squares discriminant analysis of compositional and metabonomic data. Microbial community compositional data were generated from gDNA-extracted bioreactor samples that were 16S rRNA profiled by Illumina sequencing, with subsequent processing and center log-ratio transformation, and from ^1^H NMR metabolite data from filtered (0.2-μm pore size) bioreactor samples. The bioreactors were seeded with a defined microbial community representing a fecal sample from an ulcerative colitis patient. Data points are labeled by the following sample characteristics: (i) day of the bioreactor run (D), (ii) replicate number (R), and (iii) the assignment to rifaximin treatment (C for control and T for treatment). Coloring is used to distinguish the experimental groups, as indicated. Before, samples collected prior to rifaximin treatment or application of MET; Abx, rifaximin pretreatment.

**FIG 2:**
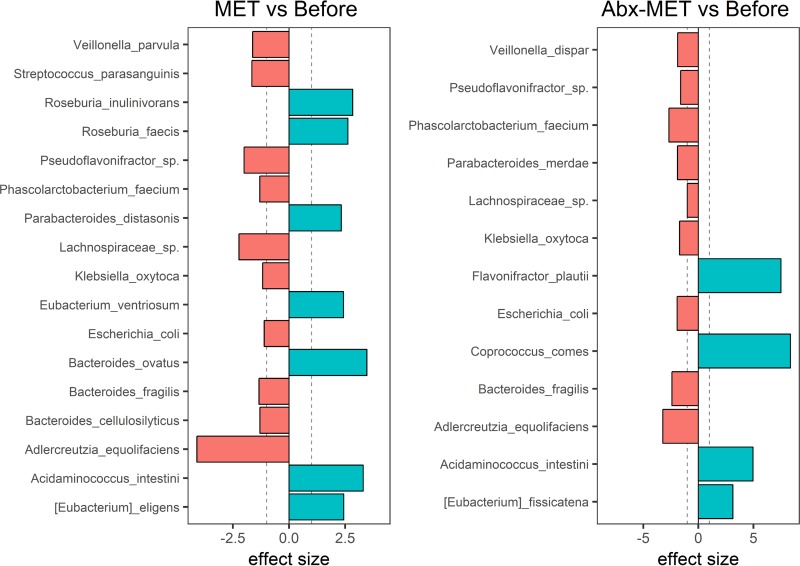
Differential abundance changes in the ulcerative colitis-associated microbial community after microbial replenishment by rifaximin pretreatment. Pairwise effect sizes of processed and center log-ratio-transformed Illumina 16S rRNA profiling data were generated from gDNA-extracted bioreactor samples seeded with a defined microbial community representing a fecal sample from an ulcerative colitis patient. Shown are the species with absolute effect sizes greater than 1, when abundances before treatment (Before) and after microbial ecosystem therapeutic replenishment conditions, both untreated (MET) and pretreated with rifaximin (Abx-MET), were compared. Dotted lines are at the −1 and 1 effect sizes for reference.

To determine the effects of rifaximin pretreatment on FMT into a UC-associated microbial ecosystem, we utilized bioreactors as *in vitro* models of the colon that were inoculated with a defined microbial community derived from a UC patient fecal sample. After establishment of the microbial communities, three replicates were pretreated with the antibiotic rifaximin, and three replicates were left untreated prior to the addition of bacterial strains derived from a healthy human fecal sample as a MET. We then carried out compositional analysis via profiling of sequenced 16S rRNA genes to elucidate which species engrafted under each tested condition. Allochthonous species that remained present after a sufficient amount of time post-therapeutic replenishment, i.e., at least 10 days under continuous culture conditions, were actively replicating and thus considered to have stably engrafted into the microbial community. However, it was also of interest to determine how the antibiotic changed the community as such alterations could provide insight into how the incoming microbes were influenced. For this analysis, genomic DNA (gDNA) was extracted from bioreactor samples that had been harvested (i) after the UC patient fecal sample-derived microbial community stabilized for 3 weeks, (ii) after three of the six bioreactor vessels were perturbed with a physiologically relevant 5-day dosage of rifaximin, and (iii) 10 to 14 days postreplenishment by MET. The overall β-diversity was significantly altered in our experiment, as assessed by permutational multivariate analysis of variance (PERMANOVA) (*P* value of 0.004) (Fig. 1). Notably, rifaximin treatment did not cause any significant shifts in relative species abundances (see Table S1 in the supplemental material). At the genus level, however, *Pseudoflavonifractor* abundance increased, with a pairwise effect size of 1.23 (*q* value of <0.0001), and *Adlercreutzia* abundance decreased, with a pairwise effect size of −2.90 (*q* value of <0.01), indicating that few alterations had occurred. Different profiles of species from MET engrafted into the control and pretreated conditions (Fig. 2; Table S1). A pairwise effect size of ≥1 was used to indicate engraftment, which is defined as the median of the differences in relative abundances between groups divided by the maximum of the median differences in relative abundances between samples within each group. By this definition, a total of seven species engrafted under the control condition, and a total of four species engrafted under the treatment condition; additionally, several members of the base community, mostly from the phylum *Proteobacteria*, experienced a decrease in relative abundance (Fig. 2). Of these species, the following were significantly changed (*q* value of <0.05): Acidaminococcus intestini, Escherichia coli, Eubacterium fissicatena, Flavonifractor plautii, Klebsiella oxytoca, *Lachnospiraceae* sp., Parabacteroides distasonis, and Parabacteroides merdae. All pairwise effect sizes, *q* values, and global effect sizes for the genus and species compositions are provided in Table S1.

10.1128/mSystems.00404-19.2 TABLE S1 Compositional data for comparing alterations in the ulcerative colitis-associated microbial community after treatments. Microbial community compositional data were generated from gDNA-extracted bioreactor samples, seeded with a defined microbial community representing an ulcerative colitis patient fecal sample, that were 16S rRNA profiled by Illumina sequencing, with subsequent processing and center-log ratio transformation. The before-treatment (Before), after antibiotic perturbation (Abx), and after microbial therapeutic replenishment with (Abx-MET) and without (MET) prior antibiotic use groups are represented, both at the species and genus taxonomic levels. Indicated are the pairwise effect sizes and one-way ANOVA with Benjamini-Hochberg corrected *P* values calculated from the rank-transformed mixed linear model-converted data, with global effect sizes as the marginal *R*^2^ value. Download Table S1, DOCX file, 0.02 MB. Copyright © 2020 Oliphant et al. 2020 Oliphant et al. This content is distributed under the terms of the Creative Commons Attribution 4.0 International license.

### Metabonomic analysis of pretreated versus untreated communities revealed differences in saccharolytic and proteolytic fermentation.

Metabonomic profiles derived from ^1^H nuclear magnetic resonance (NMR) spectra provided a means of determining the behavioral changes of the defined microbial communities associated with the antibiotic perturbation and engrafted microbes, allowing greater mechanistic insights. The filtered bioreactor waste samples utilized to obtain the spectra were matched to the periods of sampling for compositional analysis. An untargeted, manual profiling approach using a standard library of compounds was applied to the spectra, and a total of 84 compounds was yielded. The metabolite concentrations that were significantly different between groupings, including before and after rifaximin application and MET replenishment under both treatment conditions, are summarized in Table 1. Pairwise effect sizes, *q* values, and global effect sizes for all metabolites are provided in Table S2. The overall metabonome was significantly altered in our experiment, as assessed by PERMANOVA (*P* value of 0.001) (Fig. 1). It was noted that rifaximin treatment resulted in significant concentration changes for several metabolites (Table 1), especially an increase in amino acids, particularly the aromatics tyrosine, tryptophan, and phenylalanine. Such alterations would indicate a selective reduction of specific fermentations involving these compounds as substrates. The metabolite concentration changes from MET replenishment were unexpectedly consistent, regardless of rifaximin pretreatment (Table 1). Both conditions resulted in a decrease of the fermentation by-products 2-hydroxyisovalerate and desaminotyrosine, a reduction of nitrogenous compounds such as glycine, isoleucine, *N*-acetylcysteine, betaine, and carnitine, and a steep decline in methanol, with concomitant increases in the fermentation by-products pyruvate and valerate. However, the untreated condition could be distinguished from the pretreatment condition through the significant reduction of sugars compared to levels in the before grouping, specifically for fructose, fucose, and galactose, suggesting a greater propensity for saccharolytic fermentation. On the other hand, the treatment condition resulted in a significant diminishing of the amino acids histidine, leucine, *N*-acetylglutamine, *N*^6^-acetyllysine, phenylalanine, and valine, which could be related to more substantive amino acid degradation capabilities.

**TABLE 1:** Differential changes in mean concentrations of metabolites after microbial replenishment by rifaximin pretreatment

Metabolite	Mean (±SD) concn by condition (mM)^*a*^				*q* value
	Before treatment	Abx	MET	Abx-MET	
2-Hydroxyvalerate	0.0218 ± 0.0074		0.0105 ± 0.0034	0.0068 ± 0.0011	0.0026
Betaine	0.2423 ± 0.0316		0.0033 ± 0.0005	0.0367 ± 0.0585	0.0090
Carnitine	0.0137 ± 0.0023		0.0045 ± 0.0024	0.0046 ± 0.0014	0.0103
Desaminotyrosine	0.0355 ± 0.0086	0.0210 ± 0.0067	0.0107 ± 0.0021	0.0101 ± 0.0024	0.0031
Fructose	0.2691 ± 0.0641		0.1752 ± 0.0390		0.0103
Fucose	0.0837 ± 0.0289		0.0371 ± 0.0052		0.0246
Galactose	0.1075 ± 0.0264	0.1661 ± 0.0223	0.0636 ± 0.0133		0.0160
Glycine	0.4420 ± 0.0959	0.7561 ± 0.1397	0.2064 ± 0.0507	0.2412 ± 0.0134	0.0013
Histidine	0.0570 ± 0.0261	0.1553 ± 0.0124		0.0233 ± 0.0111	0.0077
Isoleucine	0.2216 ± 0.0580		0.0985 ± 0.0261	0.0611 ± 0.0247	0.0026
Leucine	0.2124 ± 0.0531			0.0696 ± 0.0418	0.0235
Methanol	3.8277 ± 0.6685		0.4208 ± 0.5839	0.4626 ± 0.3068	0.0014
Methylamine	0.0220 ± 0.0098	0.0122 ± 0.0023	0.0423 ± 0.0192		0.0092
*N*-Acetylcysteine	0.1246 ± 0.0147		0.0875 ± 0.0072	0.0992 ± 0.0080	0.0103
*N*-Acetylglutamine	0.0306 ± 0.0052			0.0093 ± 0.0036	0.0064
*N*^6^-Acetyllysine	0.0772 ± 0.0204			0.0256 ± 0.0071	0.0130
Phenylalanine	0.2052 ± 0.0390	0.3170 ± 0.0871		0.0953 ± 0.0270	0.0014
Pyruvate	0.0872 ± 0.0040	0.0742 ± 0.0038	0.1780 ± 0.0255	0.1713 ± 0.0141	0.0012
Tyrosine	0.1273 ± 0.0269	0.2666 ± 0.0400			0.0023
Valerate	1.1631 ± 0.1373	0.7961 ± 0.1121	2.2932 ± 0.6921	2.2423 ± 0.2121	0.0026
Valine	0.3448 ± 0.1423			0.1042 ± 0.0430	0.0077

a Data represent ^1^H NMR-measured metabolites in 0.22-μm-pore-size-filtered bioreactor samples seeded with a defined microbial community representing an ulcerative colitis patient fecal sample that significantly changed after rifaximin treatment (Abx) or microbial ecosystem therapeutic replenishment with (Abx-MET) and without (MET) prior antibiotic usage. Only metabolites identified by Tukey’s HSD *post hoc* testing, with verification by pairwise effect sizes, from the before-treatment samples after conducting a one-way rank-transformed repeated measures ANOVA are shown.

10.1128/mSystems.00404-19.3 TABLE S2 Metabolite concentration data for comparing alterations in the ulcerative colitis-associated microbial community after treatments. Microbial community ^1^H NMR-measured metabolite data were from filtered (0.2-μm pore size) bioreactor samples seeded with a defined microbial community representing an ulcerative colitis patient fecal sample. Treatment groups represented include before treatment (Before), after antibiotic perturbation (Abx), and after microbial therapeutic replenishment with (Abx-MET) and without (MET) prior antibiotic use. Indicated are the pairwise effect sizes and one-way ANOVA with Benjamini-Hochberg corrected *P* values calculated from the rank-transformed mixed linear model-converted data, with global effect sizes as the marginal *R*^2^ value. For calculation of pairwise effect sizes, metabolites were required to be present in at least three replicates; otherwise this information is indicated as not available (NA). Download Table S2, DOCX file, 0.02 MB. Copyright © 2020 Oliphant et al. 2020 Oliphant et al. This content is distributed under the terms of the Creative Commons Attribution 4.0 International license.

### Predictive functional analysis suggested that the engraftment of allochthonous microbes into pretreated and untreated communities yielded similar contributed novel functions.

A predictive functional analysis was conducted to not only determine which capabilities were provided by the engrafted microbes but also to provide a putative link for the compositional and metabonomic results. KEGG orthologies (KO) were obtained from the available genomic data of all species present in the bioreactors, as defined by 0.01% compositional abundance in at least three replicates by 16S rRNA gene Illumina sequence profiling, which included bacteria from both the UC patient fecal sample-derived defined microbial community and MET. A total of 53.8% of the 26 species present had genomes obtained from KEGG, 34.6% had genomes found in NCBI, and 11.6% had draft genomes constructed from shotgun genomic Illumina sequencing data of the strains (Table S3). A range of 1 to 26 genomes could be attained per species, with only one species requiring use of deposited proteome data in the absence of a complete genome through NCBI (Table S3). The top three KEGG pathways attributed to each species by unique KOs that engrafted under both rifaximin-pretreated and untreated conditions are described in Table 2. A few added predicted functionalities were noted, particularly the heightened capacity for the utilization of amino sugars and nucleotide sugars, carbohydrates, and select amino acids. The *Lachnospiraceae* spp. especially contributed these carbohydrate-utilizing functionalities, with a higher functional potential observed under the condition without prior rifaximin use due to the increased amount of incorporation from this family. Another distinguishing feature between conditions was the preference of specific amino acid substrates, with the untreated condition favoring arginine and proline metabolism by Bacteroides ovatus and P. distasonis and the rifaximin-pretreated condition favoring lysine degradation by F. plautii.

**TABLE 2:** Contribution of MET to KEGG metabolic pathways

Pathway	No. of novel orthologies by condition and species^*a*^									
	All	MET						Abx-MET		
	*A. intestini*	*E. eligens*	*B. ovatus*	*E. ventriosum*	*P. distasonis*	*R. faecis*	*R. inulinivorans*	*E. fissicatena*	*C. comes*	*F. plautii*
Amino sugar and nucleotide sugar metabolism		+		+		++	+			+
Arginine and proline metabolism		+	+		++	+	+	+		+
Butanoate metabolism	+			++++				++		+++
d-Arginine and d-ornithine metabolism										++++
Fructose and mannose metabolism			+							+
Galactose metabolism					+	+		++	+	
Glycerophospholipid metabolism		+		+	+		+	+	+	+
Lysine degradation										++++++
Methane metabolism				++++				+++		+
Propanoate metabolism				++++				++		
Riboflavin metabolism			+							++
Starch and sucrose metabolism		+		+	+	+++	++	++	++	
Sulfur metabolism		++				++				

a Data represent the top three KEGG metabolic pathways that were attributed to each engrafted species from the healthy consortium of microbes (MET) into the ulcerative colitis-associated microbial community with and without rifaximin pretreatment (Abx), as determined by the number of KEGG orthologies contributed by each allochthonous species that were novel to the ulcerative colitis-associated microbial community present in the bioreactors. Each of these novel KEGG orthologies is represented by a + symbol, so that the number of novel KEGG orthologies per KEGG metabolic pathway may be observed. Species represented are the following: Acidaminococcus intestini, Eubacterium eligens, Bacteroides ovatus, Eubacterium ventriosum, Parabacteroides distasonis, Roseburia faecis, Roseburia inulinivorans, Eubacterium fissicatena, Coprococcus comes, and Flavonifractor plautii.

10.1128/mSystems.00404-19.4 TABLE S3 Genome information for all species present in the bioreactors. Data shown are source, condition, source of genomes, and number of genomes from which proteome data were obtained for each species present in the bioreactors, as defined by at least 0.01% compositional abundance in three replicates from 16S rRNA profiling via Illumina sequencing. The species were sourced from defined microbial communities, either derived from an ulcerative colitis patient fecal sample (UCC) or a healthy-donor fecal sample (MET). The UCC species were present both before and after treatments (All), whereas the MET species either engrafted under the untreated condition (MET) or under the rifaximin-pretreated condition (Abx-MET). Genomes were obtained from KEGG or NCBI. When deposited proteome data could be obtained from NCBI despite the absence of a complete genome, the genome source is listed as NCBI, but the number of genomes is listed as zero. If no data were available, either due to the strain being a potential novel species or otherwise, a draft genome of the strain was assembled and annotated from shotgun genomic Illumina sequencing conducted at the Broad Institute. Download Table S3, DOCX file, 0.01 MB. Copyright © 2020 Oliphant et al. 2020 Oliphant et al. This content is distributed under the terms of the Creative Commons Attribution 4.0 International license.

## DISCUSSION

There is currently great interest in using FMT strategies to treat GI disorders, and therefore gaining insights into how to improve the incorporation of therapeutic microbes into patient microbial communities is key for the development of this treatment modality. One of the considerations for the use of FMT is whether antibiotic therapy should be applied to a patient prior to the addition of beneficial microbes, with the rationale that incoming bacterial species may be better able to utilize a vacated rather than an occupied niche. In this study, we attempted to address the question of whether niche availability might influence introduced species engraftment by treating a UC-derived microbial community with rifaximin before introduction of our healthy-donor-derived strains. Rifaximin was selected because it is known to have beneficial effects as a treatment for reducing the severity of UC flares (17–20), and it has previously been found that using this antibiotic as a pretreatment improved the secondary outcomes of patients after FMT compared to results in untreated controls (16). We chose to focus on microbial ecosystem changes mimicking the introduction of healthy-donor-derived microbes into a defined UC-derived community using an *in vitro* model of the colonic microbiota. This approach allowed us to determine species-level interactions in a reproducible and highly detailed way that we hope may offer a foundation for development of rationally designed microbial ecosystem therapeutic strategies for the treatment of GI disorders such as UC.

Several studies have demonstrated that rifaximin treatment has the potential to modulate gut microbial communities in dysbiotic ecosystems; however, overall composition is often not apparently altered (21–23). In this work, we also found that the effect of the antibiotic on the relative species abundances in our UC patient fecal sample-derived microbial community was minimal (see Table S1 in the supplemental material). Although our experimental design used a defined community, perhaps limiting the effects of antibiotic perturbation, the benefit of this reductionist approach was that we were clearly able to discern significant relative abundance shifts in a few taxa, such as *Pseudoflavonifractor* sp. and Adlercreutzia equolifaciens (Table S1). These taxa did follow the expected trends based on the antibiotic resistance profiles (Table S4); however, it is still surprising that more of the relatively resistant strains did not proliferate in this situation. As rifaximin is considered to have both bactericidal and bacteriostatic properties, the latter ability to limit behaviors conducive to proper growth may be key to its beneficial attributes.

10.1128/mSystems.00404-19.5 TABLE S4 Bacterial strain information and relative rifaximin resistance for the defined microbial communities. Bacterial strain source, taxonomic information, relative abundance in formulation, and rifaximin resistance profiles of the defined microbial community derived from an ulcerative colitis patient fecal sample (UCC) and the microbial ecosystem therapeutic (MET) are shown. Strains that were not originally included in the formulation described in Petrof et al. (51) are indicated as MET-A. Closest species matches were determined from NCBI BLAST searches of the 16S rRNA gene, with matches with <97% identity denoted by the genus, and those with <95% identity denoted by the family. The relative abundance in formulation is represented by biomass measured using standard 10-μl loopfuls. Note that relative abundance applies only to MET as the UCC strains were added in equal ratios since the community was allowed ample equilibration time in the bioreactor. Rifaximin resistance profiles were determined via the disk diffusion method with a 40-μg dosage. The diameters were measured in centimeters and assigned to categories as follows: completely resistant (R), highly resistant, 1 to 2 cm (+); relatively resistant, 2 to 3 cm (++), sensitive, 3 to 4 cm (+++), and very sensitive, 4+ cm (++++). Download Table S4, DOCX file, 0.01 MB. Copyright © 2020 Oliphant et al. 2020 Oliphant et al. This content is distributed under the terms of the Creative Commons Attribution 4.0 International license.

Indeed, in contrast to the few changes in relative abundances of taxa noted above, we observed a significant change in several metabolite concentrations after rifaximin treatment (Table 1). Rifaximin’s mechanism of action lies in its ability to inhibit bacterial DNA-dependent RNA polymerase, and thus it is not unexpected that specific bacterial metabolisms are reduced through treatment with this drug. We detected both increases of certain amino acids and sugars and decreases in their fermentation by-products in the UC patient fecal sample-derived community treated with rifaximin compared to levels in the untreated community (Table 1). Particularly interesting were the increases in the aromatic amino acids histidine, phenylalanine, and tyrosine as compounds that can be produced from their fermentation, such as histamine, tyramine, phenol, and *p*-cresol, may have detrimental effects on the host (2). Other groups of investigators have reported similar changes in amino acids that can yield harmful by-products after rifaximin treatment; Kang et al. determined that less glutamine was converted to glutamate and ammonia in mouse models of minimal hepatic encephalopathy (24), and Maccaferri et al. also found that tyrosine increased in their bioreactor experiments modeling Crohn’s disease (21). Together, our data fit with the hypothesis that rifaximin has a greater effect as a modulator of microbial behavior than as an antibiotic and that its beneficial attributes as a treatment for UC flares may be more related to its ability to regulate host-detrimental metabolism or the expression of virulence factors (25). However, limitations of our model mean that any direct effects of rifaximin on the host immune system or on bacterial adhesion to the host epithelium could not be tested, and these may also be important in the management of disease (24–27).

When we used rifaximin as an antibiotic pretreatment of our UC patient feces-derived community prior to the introduction of healthy donor-derived bacterial species, we saw a pronounced effect on the engraftment of allochthonous microbes into the UC community compared to that of the untreated condition (Fig. 2). Our calculations estimated that only 3% of the maximum concentration of rifaximin, or 21 μg/ml, would have been retained at the second time point of MET delivery (Fig. S1), and this value is likely inflated as it does not account for degradation of the antibiotic (for example, through exposure to light). Yet this small amount may still have been a driver of engraftment abilities as we found that members of the healthy donor-derived ecosystem that were very sensitive to rifaximin, indeed, did not engraft, for example *Roseburia* spp., Eubacterium eligens, and Eubacterium ventriosum (Fig. 2; Table S4). The notable exception to this pattern was that the relatively resistant strain of B. ovatus did not integrate into the treated community yet did integrate into the untreated community. KO analysis suggested that the contributions of B. ovatus to the UC MET community were related to riboflavin, arginine and proline, and fructose and mannose metabolism (Table 2). As *Pseudoflavonifractor* sp. (UC community member) and F. plautii (MET community member) are also capable of performing these metabolisms and as strains of these species were found to be abundant under the condition with rifaximin pretreatment, B. ovatus may have been outcompeted in this situation.

10.1128/mSystems.00404-19.1 FIG S1 Calculated rifaximin concentration in the bioreactors over time. The dosing regimen of 100 mg every 12 h into 400-ml vessels with a medium feed rate equivalent to 400 ml/day was begun on day 21 of the run and ended on day 25. The time points at which the microbial ecosystem therapeutic was pulsed into the bioreactors is indicated by the dotted line, at days 27 and 29. The maximum concentration of rifaximin reached was 672.86 μg/ml, and the amounts retained on delivery days peaked at 156.52 μg/ml and 21.18 μg/ml, respectively. Download FIG S1, JPG file, 0.2 MB. Copyright © 2020 Oliphant et al. 2020 Oliphant et al. This content is distributed under the terms of the Creative Commons Attribution 4.0 International license.

There were few notable differences in the metabonomes of the rifaximin-pretreated and untreated conditions after MET replenishment, however. A greater number of amino acids decreased in concentration following MET addition into the antibiotic-pretreated condition, and a greater number of sugars decreased in concentration following MET addition into the untreated condition (Table 1). It is possible that the former difference could be attributed to the multiple KOs of the rifaximin-resistant strain, F. plautii, that were related to the degradation of several amino acids, as determined by the predicted functional analysis (Table 2). The latter heightened capacity for dietary carbohydrates was linked by KOs to the primary degraders from the phylum *Bacteroidetes* and *Lachnospiraceae* spp. Particularly, fucose, a component of mucin (28), may have been associated with an enhanced ability for mucin glycan foraging provided by the greater number of *Lachnospiraceae* members that incorporated into the untreated condition. Overall, both the metabonomic and predictive functional analyses suggest that, despite distinct profiles of species integrating under each experimental condition, similar ecological niches were filled (Tables 1 and 2).

Ultimately, we found that both the rifaximin-pretreated and the untreated conditions resulted in engraftment of bacterial strains representing species from *Clostridium* clusters IV and XIVa, together with a concomitant decrease in *Proteobacteria* spp. (Fig. 2). Both of these outcomes (and in particular the increase of *Clostridiales* taxa associated with butyrate production) are associated with improved clinical response rates to FMT in UC patients (29–35). While the introduced MET treatment changed the metabolism of the patient fecal sample-derived community, we noted that most measured metabolite concentrations in our study were not influenced by rifaximin pretreatment (Table S2). For example, methanol, 2-hydroxyisovalerate, betaine, and carnitine, compounds that are associated with poor health outcomes under elevated conditions (36–41), all significantly decreased following MET introduction into the UC community, either with or without rifaximin pretreatment (Table 1). In an analogous clinical study, El-Nachef and colleagues also found no significant difference in the primary endpoints between rifaximin-pretreated and untreated patients (16). Rather, it is possible that their observed positive secondary outcomes are related to the behavioral alterations induced by the antibiotic itself as shown in our and other studies (21, 24, 25); the effect of the antibiotic alone was not included as a control.

El-Nachef et al. proposed that a combination of bactericidal broad-spectrum antibiotics administered prior to FMT may be necessary to provide the desired outcome (16). Indeed, Ji et al. found that a cocktail of ampicillin, neomycin, and vancomycin promoted colonization of allochthonous microbes into specific-pathogen-free mice (42). In this case, the authors concluded that the proposed hypothesis of antibiotics clearing niches to allow such colonization to occur was correct. However, the results of our study contrast with this finding as we found that more bacterial strains integrated into the untreated than into the treated UC community. There are two possible reasons for this discrepancy. First, Ji et al. utilized healthy mice, in which the resident gut microbial ecosystems are not expected to be representative of the diseased state (42). In contrast, and as an example, UC patients are depleted in microbial diversity, particularly in members of the *Lachnospiraceae* and *Ruminococcaceae* families (43). Moayyedi et al. did not pretreat UC patients with antibiotics prior to FMT and found that the bacterial family *Lachnospiraceae* and the genus *Ruminococcus* were associated with their donor that successfully induced remission (44), indicating that UC patients can be innately primed to accept beneficial bacteria unlike healthy individuals. However, a more important consideration may be the ability of the incoming microbes to survive in the antibiotic-treated environment. Kump et al. pretreated UC patients with vancomycin, paromomycin, and nystatin prior to FMT and determined that the bacterial family unclassified *Ruminococcaceae* and the genera *Akkermansia* and *Ruminococcus* were associated with their donors that successfully induced remission (45). Similarly, the *in vitro* work we describe here demonstrated that many *Lachnospiraceae* spp. integrated into the untreated microbial community, and F. plautii, an unclassified *Ruminococcaceae* family member, significantly engrafted following antibiotic pretreatment. In the KEGG database, a representative F. plautii genome (KEGG accession no. T04429) possesses multiple complete modules related to vancomycin, lantibiotic, fluoroquinolone, and bacitracin resistance (46), suggesting that it and perhaps related taxa may be more suited to surviving within an antibiotic-pretreated environment. In contrast, members of the *Lachnospiraceae* family are highly sensitive to antibiotics such as vancomycin (45, 47) and rifaximin (this work). Our findings therefore do appear to match studies of human trials of FMT for UC treatment that measured changes in the gut microbiota of the recipients, despite the difference in antibiotics used.

In conclusion, the recommendation of Keshteli et al. that antibiotic pretreatment could improve FMT efficacy in UC patients was not supported by this study from the perspective of using rifaximin as the pretreatment (11). Instead, we would suggest that future work investigates the use of a selective bactericide rather than a broad-spectrum antibiotic. This strategy would be more directed against opportunistic pathogens that are occupying critical niches and would simultaneously reduce the disturbance of beneficial bacteria, which our study demonstrated to be a crucial factor of successful colonization. For example, an effective tactic for UC might be pretreatment with an antibiotic active against *Proteobacteria*, such as ceftazidime, which could then allow more *Lachnospiraceae* and *Ruminococcaceae* to colonize (43, 48). Such targeted approaches are already yielding improvements in the clinical setting; for example, Thorpe et al. had a reduced rate of recurrence of Clostridioides difficile infection in their patients after using the antibiotic ridinilazole, which was active against the pathogen but spared *Lachnospiraceae* spp., compared to the rate of traditional vancomycin therapy (47). As we have demonstrated that our *in vitro*, bioreactor-based, colonic ecosystem model produces comparable results to literature reports of clinical work, we would suggest it as a useful tool for screening these selective antibiotics and, thus, the effectiveness of this strategy.

## MATERIALS AND METHODS

### Bioreactor operation and defined microbial communities.

A Multifors bioreactor system (Infors AG, Bottmingen/Basel, Switzerland) was operated as an *in vitro* model of the distal human gut with a working volume of 400 ml, as described previously (49). Briefly, bioreactors were run under the following conditions to mimic the physiological conditions found within the human colon: (i) 37°C, (ii) pH 7.0, (iii) retention time of 24 h (400 ml of feed added per vessel per day at a constant rate while maintaining volume), and (iv) anaerobic conditions through sparging of N_2_ gas. The bioreactor medium formulation was as stated for replicate one of this study. Thereafter, a single component of the formulation, xylan (Sigma-Aldrich, Saint Louis, MO, USA), was replaced by xylooligosaccharide (BioNutrition, Laval, QC, Canada) because of discontinuation of the xylan product; however, this formulation change was not found to affect ecosystem function or dynamics by partial least squares discriminant analysis (Fig. 1). Bioreactor vessels were inoculated with a defined microbial community that comprised 24 bacterial strains originally derived from the fecal sample of a UC patient, and our previous work has shown that this community is able to heighten the TH_17_ response and increase the sensitivity to colitis in mice, as characteristic of the disease (see Table S4 in the supplemental material) (50). Bioreactors were allowed 3 weeks to equilibrate (49). Three replicate bioreactors each were assigned to the conditions of control and treatment in a randomized fashion, for a total of six. Control bioreactors were administered 10 ml of the MET in a relative ratio as described in Petrof et al. (51), and additional isolates were included to improve the metabolic diversity of the formulation (Table S4). Rifaximin resistance profiles for all strains were analyzed by a Kirby-Bauer disk diffusion test using disks impregnated with 40 μg of rifaximin as described in Huhulescu et al. (52), prior to the bioreactor experiments. Treatment bioreactors were perturbed with the clinically relevant dosage of 200 mg/day rifaximin (Sigma-Aldrich) (17), which was used with equivalence *in vitro* since the antibiotic is not gut permeable (<0.4% systemically absorbed) (53). As such, the vessels were pulsed with 100 mg, using 5 ml of ethanol as a carrier, every 12 h for 5 days, prior to the administration of MET. Under this condition, MET was added twice, at 2 days and 4 days after rifaximin treatment to increase the probability of capturing the point at which the underlying microbial ecosystem had not fully recovered from the perturbation yet enough of the residual antibiotic had been removed to lessen the disturbance of incoming microbes. The hypothetical amount of rifaximin remaining at these time points was calculated, through use of the R package deSolve (version 1.21), which determined that roughly 23% of the maximum concentration would have been retained at the first time point, and 3% would have been retained at the second time point (Fig. S1). Sampling was conducted at time points directly after equilibration, directly after the course of antibiotics, and 10 to 14 days after MET replenishment.

### 16S rRNA compositional profiling.

A QIAamp Fast DNA Stool minikit (Qiagen Inc., Germantown, MD, USA) was utilized according to the manufacturer’s directions to extract gDNA from the cellular pellet of 2 ml of culture per bioreactor sample. 16S rRNA libraries were prepped with 400 ng of Nextera XT Index V2 sequences (Illumina, Inc., Hayward, CA, USA) plus standard V4 region primers (54) and 2 μl of gDNA template in Invitrogen Platinum PCR SuperMix High Fidelity (Life Technologies, Burlington, ON, Canada) as a one-step PCR amplification. Cycler conditions included an initial melting step of 94°C for 2 min, followed by 50 cycles of 94°C for 30 s, annealing temperature for 30 s, and 68°C for 30 s, with a final extension step of 68°C for 5 min. The annealing temperature was comprised of a 0.5°C increment touchdown starting at 65°C for 30 cycles, followed by 20 cycles at 55°C. PCR products were purified using an Invitrogen PureLink PCR purification kit (Life Technologies) according to the manufacturer’s directions. Subsequent normalization and Illumina MiSeq sequencing were carried out at the Advanced Analysis Center located in the University of Guelph, Ontario, Canada. Sequencing data were processed in R (version 3.4.4) according to the recommended procedure of the package DADA2 (55), version 1.6, with classification to the genus level by the SILVA database (56), version 132 (https://benjjneb.github.io/dada2/training.html). Amplicon sequencing variants (ASVs) were next classified to the species level by first identifying the top hits of NCBI BLAST searches (https://blast.ncbi.nlm.nih.gov) via percentage identity and E value and then cross-referencing with the known members of the defined microbial communities to determine the correct identification. ASVs that classified to redundant species were amalgamated so that each ASV was attributed to a unique species, and those that did not represent 0.01% total abundance in at least one sample were removed. Similarly, a set of ASVs at the genus level was created by amalgamating ASVs that were classified to the same genus. Finally, the sequencing data were center log-ratio transformed by the package ALDEx2 (57), version 1.10, taking the median of the Monte Carlo instances as the value for relative abundance.

### ^1^H NMR metabonomics.

Sample preparation, ^1^H NMR spectral acquisition and processing, and profiling of metabolites were conducted as previously described (49). Briefly, samples were centrifuged at 14,000 rpm (maximum speed) for 15 min to clarify, and the supernatants were passed through a 0.22-μm-pore-size filter. The addition of Chenomx internal standard (Chenomx, Inc., Edmonton, AB, Canada) to the filtrates at 10% (vol/vol) and the scanning parameters were implemented according to the recommendations of the Chenomx NMR suite, version 8.1, in order to match the found compounds to those in the library. A Bruker Avance III 600.00 MHz spectrometer with a TCI 600 probe (Bruker, Billerica, MA, USA) at the Advanced Analysis Center located in the University of Guelph, ON, Canada, was utilized for spectral acquisition. Spectra were collected at a sample temperature of 295 K. The data were analyzed using both untargeted spectral binning and metabolite profiling with the Chenomx NMR suite. For spectral binning, the default parameters of a bin size of 0.04 ppm along the 0.04- to 10-ppm region of the spectrum line with omission of water (4.44 to 5.50 ppm) and normalization by standardized area (fraction of the chemical shape indicator, 4,4-dimethyl-4-silapentane-1-sulfonate [DSS]) were implemented. Metabolite profiling was conducted utilizing the internal library of compounds from which identifications were based on the best fit for the peak regions.

### Statistical analysis.

To determine which species and metabolites were significantly different, a series of one-way tests was conducted for each data set by first rank transforming the data due to its nonparametric nature and then converting it to a mixed linear model to handle its dependent and unbalanced attributes to complete an analysis of variance (ANOVA). The *P* values were subsequently adjusted via the Benjamini-Hochberg method to correct for multiple testing (i.e., to obtain *q* values). Effect sizes were calculated as the marginal *R*^2^ value from the model. Only features that both had an effect size of above 50% and were under a *q* value threshold of 0.05 were considered significant. Groupings of significant features were determined from Tukey’s honestly significant differences (HSD) *post hoc* tests, which were verified by calculating pairwise effect sizes (58). For compositional data, species unique to the MET with high pairwise effect sizes were also considered, even in the absence of statistical significance, which was a result of these species attaining a lower relative abundance in the final community structure but did not preclude their presence. Partial least squares discriminant analysis was used to visualize how these significantly different features (i.e., biomarkers) separated the data. In the case of the metabonomics data set, the spectral binning data were used so that unprofiled compounds could also be observed. The significance of these models was verified by permutation testing utilizing the *R*^2^ and *Q*^2^ quality metrics (59). Additionally, PERMANOVA with permutations constrained within bioreactors was used on Euclidean distance matrices computed from these data sets to evaluate whether or not the visualized separations were significant (60). All analysis was conducted in R with use of the packages lme4 (version 1.1.17), lmerTest (version 3.0.1), MuMIn (version 1.40.4), lsmeans (version 2.27.62), ropls (version 3.7), and vegan (version 2.5.6). Figures were generated by the package ggplot2, version 2.2.1.

### Functional and pathway analysis.

To gain insight into the connection between condition and taxonomic and metabolic changes, protein sequences derived from fully sequenced genomes were first obtained for each species present in the bioreactors, as defined by 0.01% compositional abundance in at least three replicates from 16S rRNA profiling via Illumina sequencing. The KEGG database (https://www.genome.jp/kegg) was first utilized, and if no genomes were available for a given species, the NCBI genome database (https://www.ncbi.nlm.nih.gov/genome) was used (Table S3); genome retrieval from the two databases was automated with use of R packages KEGGREST, version 1.20, and rentrez, version 1.2.1, respectively. If protein data were available for a given species in the absence of a complete genome through NCBI (https://www.ncbi.nlm.nih.gov/protein), this deposited data were used instead (Table S3). For three of the bacterial strains, *Lachnospiraceae* sp., Phascolarctobacterium faecium, and *Pseudoflavonifractor* sp., genomes of exact species could not be obtained from these databases. Draft genomes were thus *de novo* assembled from shotgun genomic Illumina sequencing data obtained from the Broad Institute (Cambridge, MA, USA) and deposited in NCBI (https://www.ncbi.nlm.nih.gov/taxonomy), via the Shovill pipeline, version 0.2 (https://github.com/tseemann/shovill), which uses the SPAdes algorithm (61), version 3.12, with subsequent annotation by Prokka (62), version 1.12 (Table S3). KO annotation was either provided directly if the genome could be acquired from KEGG or was conducted via the online tool GhostKOALA (https://www.kegg.jp/ghostkoala). The KOs that were unique to the species that engrafted were then obtained for each condition, with and without rifaximin pretreatment. KOs were then linked to their respective KEGG pathways, and the number of unique KOs attributed to each pathway by organism was tabulated.

### Data availability.

The raw sequencing data generated during the current study is available in the NCBI repository under BioProject accession number PRJNA488265.
