# Supplementary material for: Effects of Antibiotic Pretreatment of an Ulcerative Colitis-Derived Fecal Microbial Community on the Integration of Therapeutic Bacteria In Vitro
Source: mSystems. 2020 Jan 28;5(1):e00404-19. doi: 10.1128/mSystems.00404-19 (PMC6989129; doi:10.1128/mSystems.00404-19)
Supplement: TABLE S1 [file mSystems.00404-19-st001.docx]

| **Species** | **Abx vs. Before effect size** | **MET vs. Before effect size** | **Abx-MET vs. Before effect size** | **Abx-MET vs. Abx effect size** | **q-value** | **Global effect size** |
| --- | --- | --- | --- | --- | --- | --- |
| *[Clostridium]_innocuum* | -0.03 | -0.69 | -0.64 | -0.44 | 0.0537 | 0.43 |
| *[Eubacterium]_eligens* | 0.07 | 2.44 | 0.95 | -0.07 | 0.0352 | 0.47 |
| *[Eubacterium]_fissicatena* | -0.20 | 0.57 | 3.14 | 3.23 | 0.0007 | 0.73 |
| *[Eubacterium]_rectale* | 0.19 | 0.71 | 0.11 | 0.05 | 0.0631 | 0.44 |
| *Acidaminococcus_intestini* | 0.25 | 3.31 | 4.97 | 15.85 | 0.0072 | 0.69 |
| *Adlercreutzia_equolifaciens* | -3.02 | -4.11 | -3.23 | -0.16 | 0.0370 | 0.46 |
| *Akkermansia_muciniphila* | -0.31 | -0.31 | -0.23 | -0.81 | 0.2722 | 0.15 |
| *Bacteroides_cellulosilyticus* | -0.95 | -1.29 | -0.24 | 0.70 | 0.1476 | 0.32 |
| *Bacteroides_dorei* | 2.02 | -0.01 | 0.62 | -1.19 | 0.0213 | 0.51 |
| *Bacteroides_fragilis* | -2.55 | -1.34 | -2.39 | 0.81 | 0.8034 | 0.07 |
| *Bacteroides_ovatus* | 0.67 | 3.47 | 0.73 | 0.25 | 0.0118 | 0.60 |
| *Bacteroides_thetaiotaomicron* | 2.04 | -0.33 | -0.48 | -2.59 | 0.1511 | 0.19 |
| *Bifidobacterium_adolescentis* | 0.05 | -2.68 | -0.48 | -0.17 | 0.0196 | 0.35 |
| *Bifidobacterium_longum* | -0.18 | -0.10 | 0.49 | 1.53 | 0.5736 | 0.13 |
| *Bifidobacterium_pseudocatenulatum* | 0.29 | -0.17 | -0.47 | -0.30 | 0.0771 | 0.40 |
| *Blautia_luti* | 0.08 | -0.13 | -1.18 | -0.80 | 0.0464 | 0.44 |
| *Blautia_sp.* | 0.07 | -0.23 | -0.93 | -0.60 | 0.1219 | 0.34 |
| *Blautia_unclassified* | -0.45 | -0.13 | -0.70 | -0.52 | 0.1311 | 0.33 |
| *Clostridium_sensu_stricto_1*  *_unclassified_1* | -0.01 | -0.53 | -0.26 | -0.58 | 0.1409 | 0.32 |
| *Clostridium_sensu_stricto_1*  *_unclassified_2* | 0.23 | -0.11 | -0.69 | -0.85 | 0.0356 | 0.45 |
| *Clostridium_sensu_stricto_1*  *_unclassified_3* | 0.04 | -1.67 | 0.81 | -0.01 | 0.3129 | 0.19 |
| *Collinsella_aerofaciens* | -0.04 | -0.32 | -0.21 | -0.03 | 0.7682 | 0.04 |
| *Coprococcus_comes* | -0.22 | -0.27 | 8.37 | 9.74 | 0.0509 | 0.43 |
| *Dorea_formicigenerans* | -0.03 | 0.61 | -1.04 | -0.17 | 0.3969 | 0.17 |
| *Escherichia_coli* | -0.79 | -1.11 | -1.93 | -1.66 | 0.0008 | 0.72 |
| *Eubacterium_limosum* | -0.01 | -0.11 | -0.10 | -0.37 | 0.6582 | 0.06 |
| *Eubacterium_ventriosum* | -0.63 | 2.42 | -0.74 | -0.21 | 0.4639 | 0.08 |
| *Faecalibacterium_prausnitzii* | 0.12 | -0.10 | -0.04 | -0.13 | 0.1011 | 0.34 |
| *Flavonifractor_plautii* | -0.13 | -0.13 | 7.50 | 9.21 | 0.0034 | 0.64 |
| *Klebsiella_aerogenes* | -0.03 | -0.29 | -0.13 | -0.08 | 0.7943 | 0.07 |
| *Klebsiella_oxytoca* | 0.90 | -1.18 | -1.70 | -2.33 | 0.0015 | 0.72 |
| *Lachnoclostridium_sp.* | 0.57 | -0.18 | -0.20 | -0.51 | 0.0487 | 0.40 |
| *Lachnospiraceae_sp.* | -0.55 | -2.23 | -1.00 | -0.55 | 0.0088 | 0.58 |
| *Lactobacillus_casei/paracasei* | -0.06 | -0.18 | -0.22 | -0.31 | 0.2476 | 0.25 |
| *Parabacteroides_distasonis* | -0.08 | 2.32 | 0.98 | 0.95 | 0.0084 | 0.49 |
| *Parabacteroides_merdae* | -0.52 | -0.93 | -1.89 | -1.12 | 0.0024 | 0.66 |
| *Phascolarctobacterium_faecium* | -0.40 | -1.31 | -2.67 | -2.57 | 0.7355 | 0.08 |
| *Phascolarctobacterium_unclassified* | -0.05 | 0.28 | -1.01 | -0.21 | 0.5486 | 0.14 |
| *Pseudoflavonifractor_sp.* | 0.83 | -2.01 | -1.61 | -2.14 | 0.0347 | 0.43 |
| *Roseburia_faecis* | 0.01 | 2.62 | -1.00 | -0.13 | 0.0173 | 0.53 |
| *Roseburia_inulinivorans* | -0.07 | 2.84 | -0.21 | -0.23 | 0.0490 | 0.44 |
| *Ruminococcus_faecis* | 0.22 | -0.96 | 0.97 | 0.97 | 0.0044 | 0.63 |
| *Ruminococcus_unclassified* | 0.58 | -0.12 | -0.53 | -0.78 | 0.0139 | 0.55 |
| *Streptococcus_gordonii/mitis* | 0.24 | -0.22 | -0.12 | -0.20 | 0.0317 | 0.39 |
| *Streptococcus_parasanguinis* | 0.02 | -1.66 | -0.76 | -0.27 | 0.3265 | 0.14 |
| *Streptococcus_parasanguinis/gordonii* | 0.16 | -1.30 | -0.31 | -0.28 | 0.1098 | 0.35 |
| *Streptococcus_unclassified* | -0.01 | -1.31 | -0.52 | -0.22 | 0.0559 | 0.42 |
| *Sutterella_stercoricanis* | 0.43 | -0.33 | 0.86 | 0.83 | 0.0213 | 0.41 |
| *Veillonella_denticariosi* | -0.11 | -0.56 | -0.62 | -0.32 | 0.0424 | 0.30 |
| *Veillonella_dispar* | -0.19 | -0.20 | -1.87 | -0.92 | 0.0205 | 0.39 |
| *Veillonella_parvula* | 0.01 | -1.62 | -0.18 | -0.25 | 0.4399 | 0.17 |
| **Genus** | **Abx vs. Before effect size** | **MET vs. Before effect size** | **Abx-MET vs. Before effect size** | **Abx-MET vs. Abx effect size** | **q-value** | **Global effect size** |
| *[Clostridium]_innocuum_group* | -0.11 | -0.58 | -1.07 | -0.94 | 0.0298 | 0.48 |
| *[Eubacterium]_eligens_group* | 0.06 | 2.11 | 0.97 | -0.04 | 0.0584 | 0.42 |
| *[Eubacterium]_fissicatena_group* | -0.32 | 0.68 | 2.77 | 2.82 | 0.0079 | 0.48 |
| *[Eubacterium]_ventriosum_group* | 0.10 | 1.62 | -1.04 | -0.92 | 0.1256 | 0.20 |
| *[Ruminococcus]_torques_group* | 0.67 | -1.10 | 1.06 | 0.95 | 0.0450 | 0.36 |
| *Acidaminococcus* | 0.30 | 2.31 | 4.17 | 11.40 | 0.0400 | 0.46 |
| *Adlercreutzia* | -2.90 | -3.72 | -3.71 | -1.50 | 0.0030 | 0.65 |
| *Agathobacter* | 0.56 | 0.66 | -0.01 | -0.05 | 0.0057 | 0.64 |
| *Akkermansia* | -0.19 | -0.31 | -0.27 | -0.92 | 0.3638 | 0.11 |
| *Bacteroides* | 0.01 | -1.73 | -0.74 | -0.65 | 0.0265 | 0.52 |
| *Bifidobacterium* | 0.05 | -0.20 | 0.36 | 0.22 | 0.9496 | 0.02 |
| *Blautia* | 0.00 | 0.03 | -0.23 | -1.41 | 0.0576 | 0.42 |
| *Clostridium_sensu_stricto_1* | 0.01 | -0.34 | 0.20 | -0.06 | 0.6587 | 0.11 |
| *Collinsella* | -0.01 | -0.30 | -0.32 | -0.04 | 0.7546 | 0.07 |
| *Coprococcus_3* | 0.04 | -0.26 | 7.01 | 5.74 | 0.1415 | 0.32 |
| *Dorea* | -0.03 | 0.53 | -0.83 | -0.27 | 0.5744 | 0.14 |
| *Escherichia/Shigella* | -0.10 | -0.73 | -2.47 | -2.01 | 0.0048 | 0.68 |
| *Eubacterium* | -0.02 | -0.10 | -0.27 | -1.89 | 0.3013 | 0.20 |
| *Faecalibacterium* | 0.56 | -0.11 | -0.81 | -1.30 | 0.0029 | 0.65 |
| *Flavonifractor* | 0.42 | -0.08 | 11.92 | 11.66 | 0.0004 | 0.76 |
| *Klebsiella* | 0.79 | -0.63 | -4.03 | -3.97 | 0.0007 | 0.74 |
| *Lachnoclostridium* | -0.29 | -1.49 | -1.41 | -0.75 | 0.0123 | 0.58 |
| *Lactobacillus* | -0.02 | -0.31 | -0.24 | -1.10 | 0.0791 | 0.24 |
| *Parabacteroides* | -0.16 | -0.49 | -1.47 | -3.34 | 0.0088 | 0.58 |
| *Phascolarctobacterium* | 0.01 | -0.93 | -2.55 | -3.52 | 0.1120 | 0.35 |
| *Pseudoflavonifractor* | 1.23 | -1.49 | -1.63 | -2.42 | 0.0000 | 0.74 |
| *Roseburia* | 0.10 | 2.35 | -1.14 | -0.23 | 0.0408 | 0.46 |
| *Ruminococcus_2* | 0.38 | -0.07 | -0.23 | -0.87 | 0.0644 | 0.37 |
| *Streptococcus* | 0.27 | -0.31 | -0.33 | -1.23 | 0.7348 | 0.06 |
| *Sutterella* | 0.38 | -0.44 | 0.89 | 0.79 | 0.0001 | 0.37 |
| *Veillonella* | -0.36 | -0.32 | -1.50 | -1.27 | 0.0193 | 0.38 |
