# Supplementary material for: Effects of Antibiotic Pretreatment of an Ulcerative Colitis-Derived Fecal Microbial Community on the Integration of Therapeutic Bacteria In Vitro
Source: mSystems. 2020 Jan 28;5(1):e00404-19. doi: 10.1128/mSystems.00404-19 (PMC6989129; doi:10.1128/mSystems.00404-19)
Supplement: TABLE S2 [file mSystems.00404-19-st002.docx]

| **Metabolite** | **Abx vs. Before effect size** | **MET vs. Before effect size** | **Abx-MET vs. Before effect size** | **Abx-MET vs. Abx effect size** | **q-value** | **Global effect size** |
| --- | --- | --- | --- | --- | --- | --- |
| 1,3-Dihydroxyacetone | 0.35 | -0.01 | 0.21 | -0.18 | 0.6535 | 0.08 |
| 2-Hydroxyisobutyrate | 1.01 | -0.14 | -0.56 | -0.87 | 0.0942 | 0.31 |
| 2-Hydroxyisovalerate | -0.90 | -1.71 | -3.02 | -3.78 | 0.0003 | 0.77 |
| 2-Hydroxyphenylacetate | 0.11 | -0.19 | -0.22 | -0.20 | 0.0428 | 0.16 |
| 2-Oxoisocaproate | -0.29 | -0.01 | -1.01 | -0.82 | 0.5155 | 0.15 |
| 2-Phenylpropionate | 0.49 | -0.05 | -1.24 | -1.56 | 0.0661 | 0.41 |
| 3-Hydroxy-3-methylglutarate | -0.16 | -0.87 | -0.48 | -0.54 | 0.1137 | 0.15 |
| 3-Hydroxyisovalerate | 1.55 | 0.37 | 0.62 | -0.26 | 0.2181 | 0.27 |
| 3-Indoxylsulfate | -0.02 | -1.08 | -0.80 | -0.73 | 0.0637 | 0.33 |
| 4-Aminobutyrate | 4.20 | -0.09 | 0.02 | -3.72 | 0.0546 | 0.43 |
| 4-Hydroxybutyrate | 0.29 | 0.28 | -1.43 | -1.53 | 0.0404 | 0.47 |
| 5,6-Dihydrothymine | 0.39 | -0.93 | -1.15 | -1.68 | 0.0306 | 0.48 |
| 5,6-Dihydrouracil | -0.26 | -0.21 | -4.66 | -1.27 | 0.0131 | 0.43 |
| 5-Aminopentanoate | 1.44 | 0.09 | 1.83 | -0.60 | 0.0204 | 0.44 |
| Acetamide | -0.09 | -0.13 | -0.33 | -0.40 | 0.3888 | 0.14 |
| Acetate | 0.77 | 0.74 | -0.01 | -0.95 | 0.1352 | 0.32 |
| Acetoacetate | 0.45 | -0.19 | -0.62 | -1.51 | 0.1564 | 0.27 |
| Alanine | 0.50 | 0.29 | 0.02 | -0.31 | 0.7190 | 0.04 |
| Allantoin | 0.14 | -0.28 | -0.94 | -1.02 | 0.4184 | 0.18 |
| Arabinose | 1.28 | -0.38 | -0.32 | -0.90 | 0.0763 | 0.35 |
| Aspartate | -0.37 | -0.70 | -0.56 | -0.39 | 0.0499 | 0.43 |
| Betaine | 0.56 | -18.71 | -1.23 | -1.26 | 0.0015 | 0.72 |
| Butanone | 0.81 | 0.56 | -0.06 | -0.79 | 0.0083 | 0.51 |
| Butyrate | 1.57 | 2.51 | 4.58 | 5.27 | 0.0537 | 0.43 |
| Cadaverine | 0.38 | -0.09 | -0.13 | -1.12 | 0.0804 | 0.30 |
| Carnitine | 0.84 | -1.69 | -1.87 | -2.69 | 0.0022 | 0.75 |
| Carnosine | 0.78 | -0.75 | -0.38 | -1.28 | 0.1581 | 0.31 |
| Cholate | -0.06 | -0.27 | -0.65 | -0.56 | 0.5570 | 0.06 |
| Choline | 0.76 | 1.54 | 0.87 | -0.42 | 0.0138 | 0.63 |
| Creatinine | 0.09 | -1.19 | 0.23 | -0.04 | 0.1828 | 0.29 |
| Desaminotyrosine | -1.39 | -3.50 | -5.24 | -1.68 | 0.0003 | 0.80 |
| Dimethylamine | -0.01 | 0.81 | -0.07 | 0.04 | 0.8027 | 0.03 |
| Formate | 1.33 | -0.40 | -0.39 | -2.07 | 0.0018 | 0.39 |
| Fructose | 0.74 | -1.52 | -0.32 | -1.27 | 0.0023 | 0.60 |
| Fucose | 0.62 | -2.29 | -0.94 | -1.48 | 0.0067 | 0.64 |
| Fumarate | NA | 0.03 | -0.11 | NA | 0.9438 | 0.01 |
| Galactose | 1.54 | -1.67 | -0.43 | -1.21 | 0.0040 | 0.63 |
| Glucose | 0.28 | -0.45 | -0.91 | -1.76 | 0.0789 | 0.30 |
| Glutamate | -0.59 | -0.11 | -1.33 | -0.44 | 0.0737 | 0.23 |
| Glycine | 1.88 | -2.84 | -3.08 | -4.07 | 0.0000 | 0.84 |
| Glycolate | 0.83 | 0.29 | -0.68 | -1.63 | 0.0648 | 0.39 |
| Histamine | 0.65 | 0.08 | 0.25 | -0.52 | 0.0896 | 0.28 |
| Histidine | 1.76 | -0.76 | -1.09 | -2.89 | 0.0012 | 0.62 |
| Hydroxyacetone | 0.35 | -0.51 | -0.15 | -0.34 | 0.0839 | 0.32 |
| Indole-3-lactate | -0.48 | -0.56 | -0.71 | -0.30 | 0.0397 | 0.46 |
| Isocaproate | 0.36 | NA | NA | NA | 0.0547 | 0.43 |
| Isoleucine | -0.09 | -1.65 | -2.19 | -2.17 | 0.0002 | 0.73 |
| Isopropanol | 1.86 | 0.03 | 0.42 | -0.81 | 0.0353 | 0.34 |
| Isovalerate | -0.88 | -0.47 | 0.62 | 1.74 | 0.0648 | 0.22 |
| Lactate | 1.12 | -0.26 | -0.67 | -1.36 | 0.0769 | 0.39 |
| Lactose | 0.16 | 0.39 | -0.43 | -0.63 | 0.2117 | 0.26 |
| Leucine | 0.42 | -0.61 | -1.49 | -1.53 | 0.0062 | 0.53 |
| Malonate | 0.74 | 0.74 | -0.18 | -0.71 | 0.2497 | 0.16 |
| Methanol | 0.46 | -1.46 | -1.14 | -1.24 | 0.0000 | 0.68 |
| Methionine | 0.90 | -0.29 | -0.52 | -0.96 | 0.0513 | 0.38 |
| Methylamine | -1.42 | 1.13 | -0.74 | 0.39 | 0.0018 | 0.68 |
| N6-Acetyllysine | 0.20 | 1.63 | -2.47 | -2.69 | 0.0031 | 0.67 |
| N-Acetylcysteine | -0.57 | -2.84 | -1.93 | -1.18 | 0.0022 | 0.67 |
| N-Acetylglucosamine | -0.03 | -3.69 | -0.09 | -0.07 | 0.1504 | 0.32 |
| N-Acetylglutamine | -0.21 | -3.58 | -2.88 | -2.25 | 0.0008 | 0.72 |
| N-Acetylglycine | -0.23 | -0.54 | -1.24 | -0.98 | 0.0256 | 0.50 |
| Nicotinate | -0.18 | -0.49 | -0.98 | -0.79 | 0.1892 | 0.21 |
| Pantothenate | 0.01 | 0.19 | -2.99 | -0.50 | 0.0084 | 0.39 |
| p-Cresol | -0.52 | -0.22 | 0.21 | 0.43 | 0.6499 | 0.11 |
| Phenylacetate | -2.20 | -0.30 | 0.75 | 2.57 | 0.0085 | 0.48 |
| Phenylalanine | 1.04 | -0.83 | -2.59 | -3.22 | 0.0001 | 0.83 |
| Proline | 0.15 | -0.09 | -0.56 | -0.68 | 0.3132 | 0.10 |
| Propionate | 0.03 | -1.71 | -1.19 | -2.20 | 0.0140 | 0.54 |
| Pyroglutamate | -0.05 | -0.37 | -0.63 | -0.23 | 0.3033 | 0.15 |
| Pyruvate | -2.26 | 3.52 | 5.43 | 6.70 | 0.0000 | 0.84 |
| Sarcosine | 0.42 | 0.28 | -0.92 | -0.92 | 0.0006 | 0.36 |
| ß-Alanine | 0.42 | 0.70 | -0.21 | -0.51 | 0.2099 | 0.28 |
| Succinate | 0.01 | 0.19 | -0.76 | -0.35 | 0.0638 | 0.07 |
| Tartrate | 0.38 | -0.62 | -0.21 | -0.70 | 0.0346 | 0.42 |
| Thymine | -0.22 | 0.68 | 0.30 | 0.70 | 0.3290 | 0.09 |
| Trimethylamine | 0.13 | 2.68 | 0.91 | 0.79 | 0.0361 | 0.47 |
| Tryptophan | 0.26 | -0.59 | -0.15 | -0.56 | 0.2915 | 0.23 |
| Tyramine | -0.66 | -0.35 | -0.71 | 0.44 | 0.1240 | 0.23 |
| Tyrosine | 2.78 | -0.93 | -0.56 | -2.47 | 0.0001 | 0.62 |
| Uracil | 0.39 | 1.72 | -0.16 | -0.52 | 0.1593 | 0.31 |
| Urocanate | NA | NA | NA | NA | 0.3233 | 0.22 |
| Valerate | -1.40 | 1.80 | 5.30 | 4.15 | 0.0002 | 0.84 |
| Valine | 0.33 | -0.59 | -2.14 | -2.10 | 0.0012 | 0.70 |
| Xylose | 0.58 | -0.40 | -0.50 | -0.77 | 0.1008 | 0.35 |
