# Supplementary material for: Effects of Antibiotic Pretreatment of an Ulcerative Colitis-Derived Fecal Microbial Community on the Integration of Therapeutic Bacteria In Vitro
Source: mSystems. 2020 Jan 28;5(1):e00404-19. doi: 10.1128/mSystems.00404-19 (PMC6989129; doi:10.1128/mSystems.00404-19)
Supplement: TABLE S3 [file mSystems.00404-19-st003.docx]

| **Species** | **Source** | **Condition** | **Genome Source** | **Number of Genomes** |
| --- | --- | --- | --- | --- |
| *[Clostridium] innocuum* | UCC | All | NCBI | 1 |
| *Bacteroides cellulosilyticus* | UCC | All | KEGG | 1 |
| *Bacteroides dorei* | UCC | All | KEGG | 2 |
| *Bacteroides fragilis* | UCC | All | KEGG | 4 |
| *Bacteroides thetaiotamicron* | UCC | All | KEGG | 2 |
| *Klebsiella oxytoca* | UCC | All | KEGG | 3 |
| *Lachnospiraceae sp.* | UCC | All | Broad (NCBI:txid1357394) | 1 |
| *Parabacteroides merdae* | UCC | All | NCBI | 3 |
| *Phascolarctobacterium faecium* | UCC | All | Broad (NCBI:txid1357409) | 1 |
| *Pseudoflavonifractor sp.* | UCC | All | Broad (NCBI:txid1357380) | 1 |
| *Veillonella denticariosi* | UCC | All | NCBI | 1 |
| *Veillonella dispar* | UCC | All | NCBI | 2 |
| *Acidaminococcus intestini* | MET | All | KEGG | 1 |
| *[Eubacterium] eligens* | MET | MET | KEGG | 1 |
| *Bacteroides ovatus* | MET | MET | KEGG | 1 |
| *Eubacterium ventriosum* | MET | MET | NCBI | 1 |
| *Parabacteroides distasonis* | MET | MET | KEGG | 1 |
| *Roseburia faecis* | MET | MET | NCBI | 0 |
| *Roseburia inulinivorans* | MET | MET | NCBI | 1 |
| *[Eubacterium] fissicatena* | MET | Abx-MET | NCBI | 1 |
| *Coprococcus comes* | MET | Abx-MET | NCBI | 1 |
| *Flavonifractor plautii* | MET | Abx-MET | KEGG | 1 |
| *Escherichia coli* | Both | All | KEGG | 65 |
