# Supplementary material for: Effects of Antibiotic Pretreatment of an Ulcerative Colitis-Derived Fecal Microbial Community on the Integration of Therapeutic Bacteria In Vitro
Source: mSystems. 2020 Jan 28;5(1):e00404-19. doi: 10.1128/mSystems.00404-19 (PMC6989129; doi:10.1128/mSystems.00404-19)
Supplement: TABLE S4 [file mSystems.00404-19-st004.docx]

| Source | Taxonomic information | Relative abundance | Rifaximin sensitivity |
| --- | --- | --- | --- |
| UCC | *[Clostridium] innocuum* | + | + |
| UCC | *Adlercreutzia equolifaciens* | + | ++++ |
| UCC | *Bacteroides cellulosilyticus* | + | +++ |
| UCC | *Bacteroides dorei* | + | R |
| UCC | *Bacteroides fragilis* | + | ++ |
| UCC | *Bacteroides thetaiotamicron* | + | ++ |
| UCC | *Collinsella aerofaciens* | + | +++ |
| UCC | *Enterococcus faecalis* | + | ++ |
| UCC | *Escherichia coli* | + | + |
| UCC | *Klebsiella aerogenes* | + | R |
| UCC | *Klebsiella oxytoca* | + | R |
| UCC | *Lachnospiraceae sp.* | + | ++++ |
| UCC | *Parabacteroides merdae* | + | ++ |
| UCC | *Phascolarctobacterium faecium* | + | + |
| UCC | *Pseudoflavonifractor sp.* | + | + |
| UCC | *Streptococcus anginosus* | + | ++ |
| UCC | *Streptococcus gordonii* | + | ++ |
| UCC | *Streptococcus mutans* | + | +++ |
| UCC | *Streptococcus parasanguinis* | + | ++ |
| UCC | *Veillonella atypica* | + | ++ |
| UCC | *Veillonella denticariosi* | + | + |
| UCC | *Veillonella dispar* | + | ++ |
| UCC | *Veillonella parvula* | + | + |
| UCC | *Veillonella tobetsuensis* | + | ++ |
| MET | *[Eubacterium] eligens* | +++++ | ++++ |
| MET-A | *[Eubacterium] fissicatena* | +++ | ++ |
| MET | *[Eubacterium] rectale* | +++ | +++ |
| MET | *[Eubacterium] rectale* | +++ | ++ |
| MET | *[Eubacterium] rectale* | +++++ | ++++ |
| MET | *[Eubacterium] rectale* | ++++ | +++ |
| MET | *Acidaminococcus intestini* | +++ | R |
| MET-A | *Akkermansia muciniphila* | + | ++ |
| MET | *Bacteroides ovatus* | ++++ | ++ |
| MET | *Bifidobacterium adolescentis* | ++++ | ++ |
| MET | *Bifidobacterium adolescentis* | ++++ | ++ |
| MET | *Bifidobacterium longum* | + | ++ |
| MET | *Bifidobacterium longum* | +++++ | +++ |
| MET-A | *Bifidobacterium pseudocatenulatum* | +++ | ++ |
| MET | *Blautia luti* | + | + |
| MET | *Blautia sp.* | ++++ | ++ |
| MET | *Blautia stercoris* | ++ | ++++ |
| MET | *Butyricicoccus faecihominis* | +++ | ++ |
| MET | *Collinsella aerofaciens* | +++ | +++ |
| MET-A | *Coprococcus comes* | +++ | ++ |
| MET-A | *Dialister invisus* | + | ++++ |
| MET-A | *Dorea formicigenerans* | +++ | +++ |
| MET | *Dorea longicatena* | +++ | +++ |
| MET | *Dorea longicatena* | +++++ | +++ |
| MET | *Erysipelotrichaceae sp.* | +++ | + |
| MET | *Escherichia coli* | + | R |
| MET | *Eubacterium limosum* | ++++ | ++ |
| MET | *Eubacterium ventriosum* | +++ | ++++ |
| MET | *Faecalibacterium prausnitzii* | +++++ | ++ |
| MET-A | *Flavonifractor plautii* | + | R |
| MET | *Klebsiella aerogenes* | +++ | R |
| MET-A | *Lachnoclostridium sp.* | + | ++ |
| MET | *Lactobacillus casei* | +++ | + |
| MET | *Lactobacillus paracasei* | +++ | + |
| MET | *Parabacteroides distasonis* | ++++ | +++ |
| MET | *Roseburia faecis* | ++++ | +++ |
| MET-A | *Roseburia inunlinivorans* | + | ++++ |
| MET | *Ruminococcus faecis* | +++ | ++ |
| MET | *Ruminococcus faecis* | +++++ | ++ |
| MET | *Streptococcus mitis* | ++ | ++ |
| MET-A | *Sutterella stercoricanis* | + | ++ |
