# Supplementary figures and images for: Effects of Antibiotic Pretreatment of an Ulcerative Colitis-Derived Fecal Microbial Community on the Integration of Therapeutic Bacteria In Vitro
Source: mSystems. 2020 Jan 28;5(1):e00404-19. doi: 10.1128/mSystems.00404-19 (PMC6989129; doi:10.1128/mSystems.00404-19)

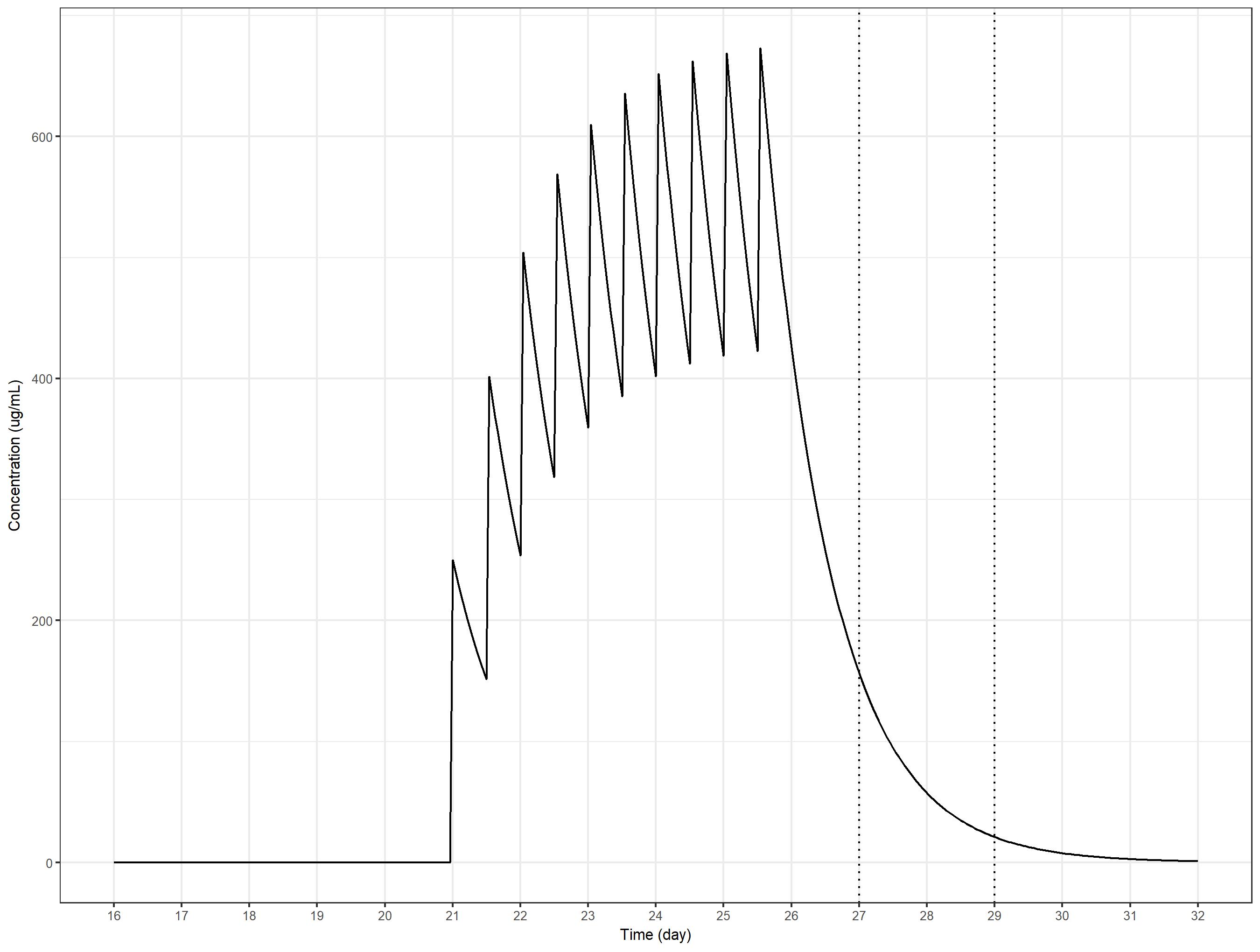

Supplement: FIG S1 [file mSystems.00404-19-sf001.jpg]
